# Supplementary material for: An Evaluation of CXCR4 Targeting with PAMAM Dendrimer Conjugates for Oncologic Applications
Source: Pharmaceutics. 2022 Mar 16;14(3):655. doi: 10.3390/pharmaceutics14030655 (PMC8953329; doi:10.3390/pharmaceutics14030655)
Supplement: Supplementary file 1 [file pharmaceutics-14-00655-s001.zip › pharmaceutics-1591814-supplementary.pdf]

pharmaceutics

Supplementary

An Evaluation of CXCR4 Targeting with PAMAM Dendrimer Conjugates for Oncologic Applications

Wojciech G. Lesniak, Babak Behnam Azad, Samit Chatterjee, Ala Lisok and Martin G. Pomper

Matrix-Assisted Laser Desorption Ionization-Time-of-Flight. Spectra were recorded on a Voyager DE-STR spectrophotometer, using 2,5- dihydroxybenzoic acid (DHB) as a matrix. Matrix and dendrimers were dissolved in 50% MeOH and 0.1% TFA aqueous solution. First, 10 μL of matrix at a concentration of 20 mg/mL was mixed with 10 μL of given dendrimer conjugate at a concentration of 4 mg/mL. Then 1 μL of the resulting mixture was placed on the target plate, evaporated and used for data collection.

Dynamic light scattering and zeta potential. Dynamic light scattering and zeta potential analyses were performed using a Malvern Zetasizer Nano ZEN3600. Results are presented as a mean of three sequential measurements. All dendrimers were prepared at a concentration of 2 mg/mL in 1xPBS.

Flow cytometry. H69 small cell lung cancer (SCLC) cells at 50-70% confluency were detached using a non-enzymatic cocktail (Gibco) and washed twice with flow cytometry buffer (1XPBS, 2mmol/L EDTA, 0.5% FBS). CXCR4 expression was determined by immunostaining with the allophycoerythrin (APC)-conjugated anti-human CXCR4 antibody (clone12G5, R&D Systems) according to the manufacturer's instructions. CXCR4 expression was analyzed on a FACSCalibur flow cytometer (Becton Dickinson). Data analysis was carried out using FlowJo software.

Inhibition of chemotaxis. The effect of G5-X4, G5-Ctrl and POL3026 on CXCR4/CXCL12-mediated chemotaxis of H69 small cell lung cancer (SCLC) cells with high CXCR4 expression was evaluated using a CytoSelect cell migration assay (Cell Biolabs, Inc. San Diego, CA) according to the manufacturer's protocol. Briefly, 50,000 cells were suspended in 100 μL RPMI containing G5-X4, G5-Ctrl and POL3026 at concentration of 10 or 100 nM and transferred into insert wells. The insert wells were placed inside the harvesting wells containing RPMI with 100 nM CXCL12 (Peprotech, Rocky Hill, NJ, product # 300-28A). After 17 h incubation at 37 °C, cells that migrated in the harvesting wells were lysed, CyQuant GR dye was added and the fluorescence was measured using Perkin Elmer-2480 Automatic Gamma Counter (PerkinElmer, Waltham, MA).

SPECT/CT imaging and analysis. For whole-body SPECT/CT imaging, NSG mouse bearing orthotopic H1155 NSCLC tumor was intravenously injected with 12.95 MBq (350 uCi) of [^{111}In]G5-Ctrl and images were acquired on an X-SPECT small animal SPECT/CT system (Gamma Medica Ideas, Northridge, CA). Prior to imaging, mouse was anesthetized with 2 % isoflurane (v/v), and maintained under 1.5 % isoflurane (v/v) during acquisition of images. Images were acquired 24, 48, 72 and 120 h after radiotracer injection using 64 projections over 360° at 45 s per projection and medium energy pinhole collimators. CT was recorded in 512 projections to allow anatomic co-registration. Data were reconstructed using the ordered subsets-expectation maximization algorithm and 3D

volume rendered images were generated using Amira 5.3.0 software (Visage Imaging Inc. San Diego, CA).

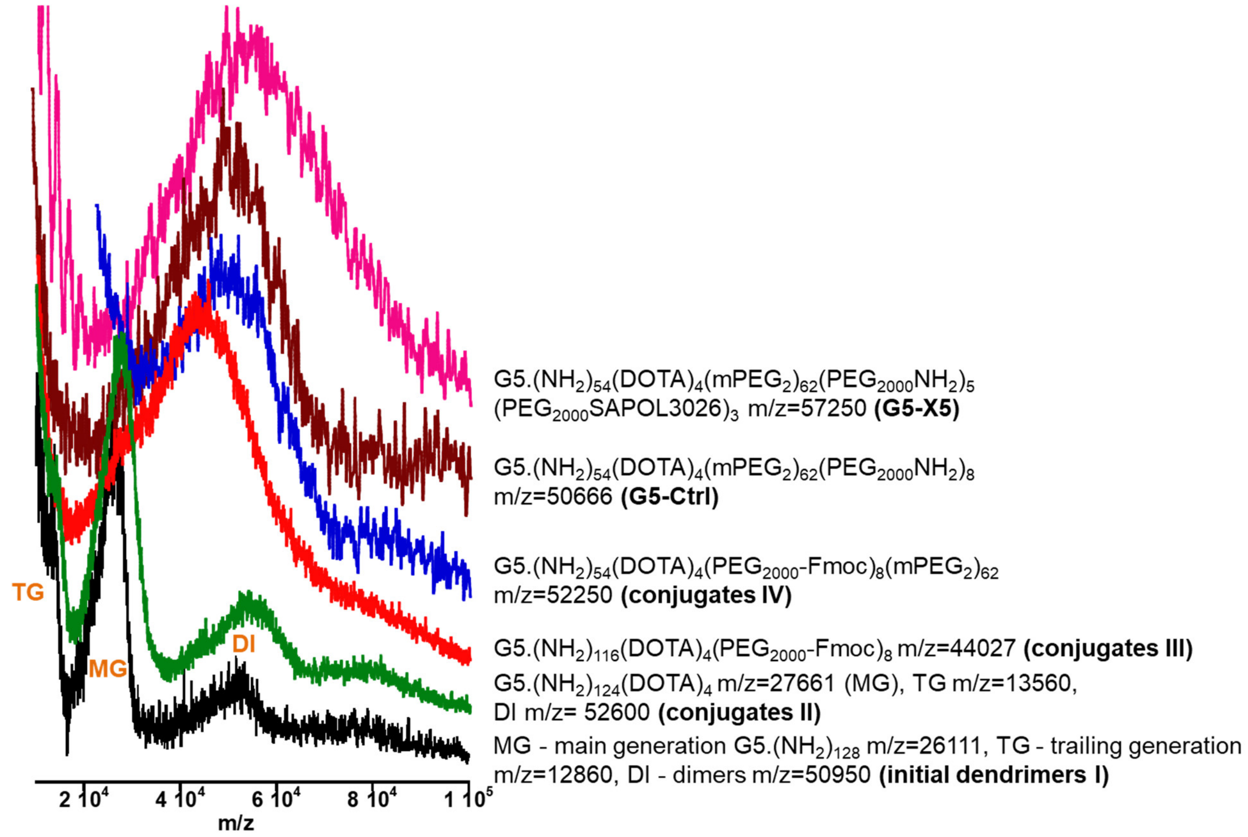

Figure S1. MALDI-TOF spectra recoded for starting dendrimers, intermediate products and final conjugates, showing changes in molecular weight upon modification step, which were used to calculated average number of conjugated moieties.

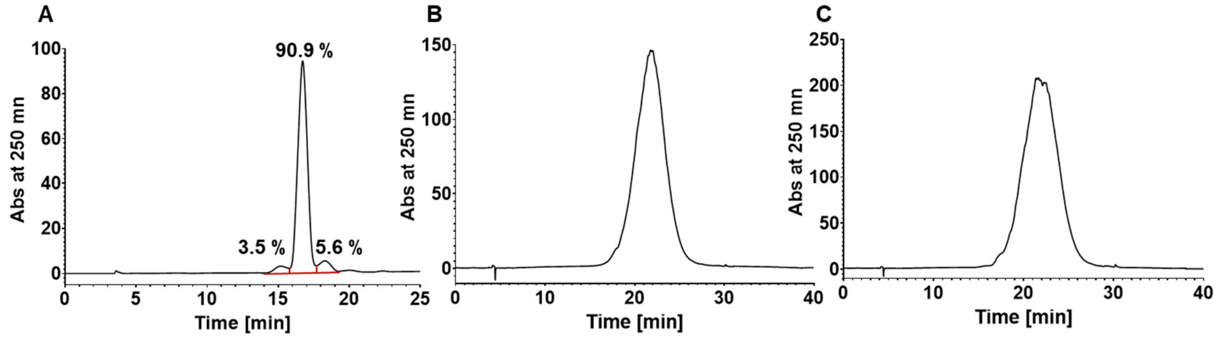

Figure S2. RP-HPLC chromatograms of: **A -** initial $G5(NH_2)_{128}$ dendrimer showing presence of trailing generation (3.5%), main generation (90.9%) and dimeric dendrimers (5.6%); **B -** G5-Ctrl and **C -** G5-X4 indicating significant widening of the peak due to presence of broad species distribution in the samples that could not be resolved. Analysis was carried out using previously reported method [1].

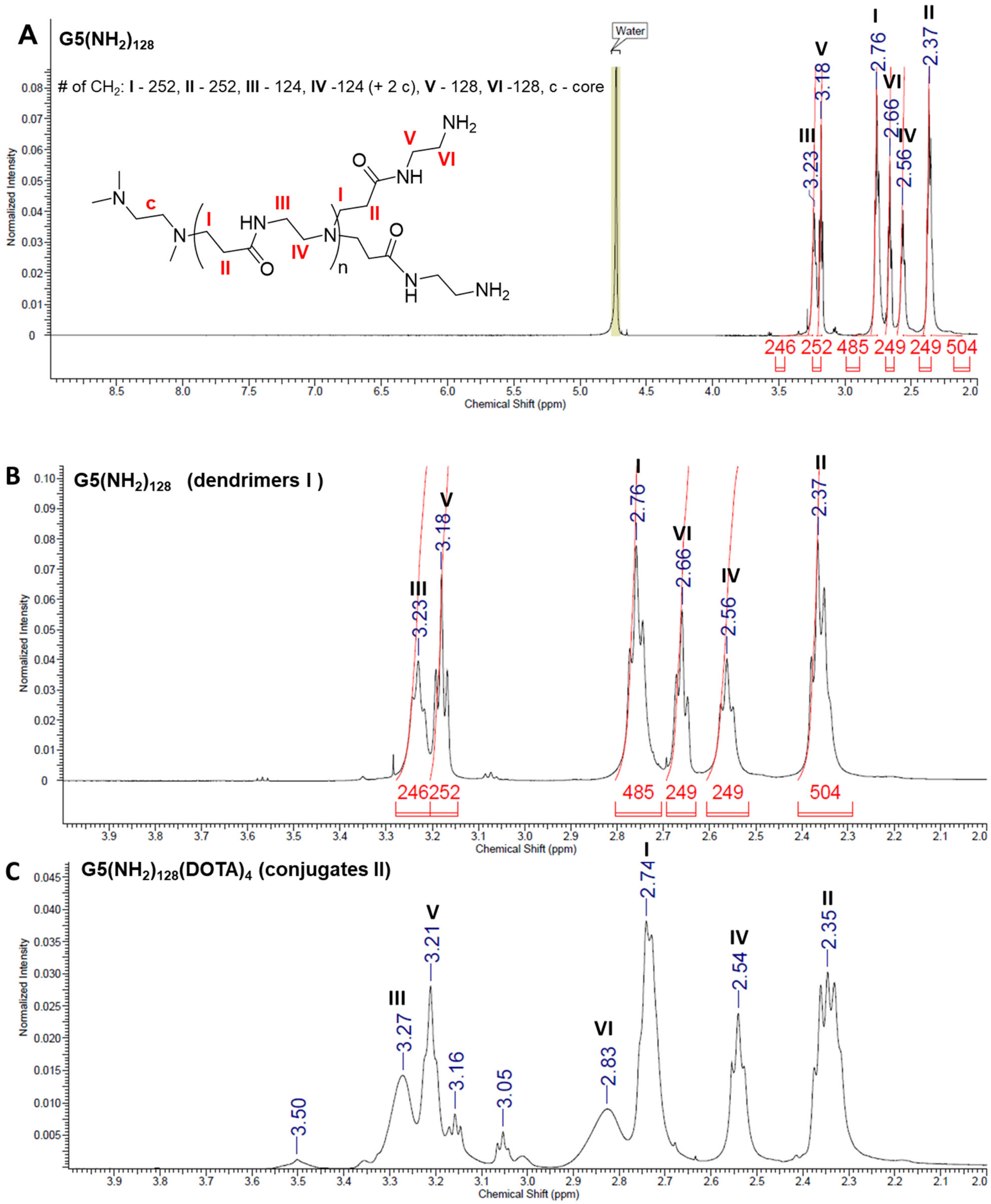
A
G5(NH2)128
# of CH2: I - 252, II - 252, III - 124, IV -124 (+ 2 c), V - 128, VI -128, c - core
Water
Normalized Intensity
Chemical Shift (ppm)
246 252 485 249 249 504
B
G5(NH2)128 (dendrimers I)
Normalized Intensity
Chemical Shift (ppm)
246252 485 249 249 504
C
G5(NH2)128(DOTA)4 (conjugates II)
Normalized Intensity
Chemical Shift (ppm)
3.50 3.27 3.21 3.16 3.05 2.83 2.74 2.54 2.35

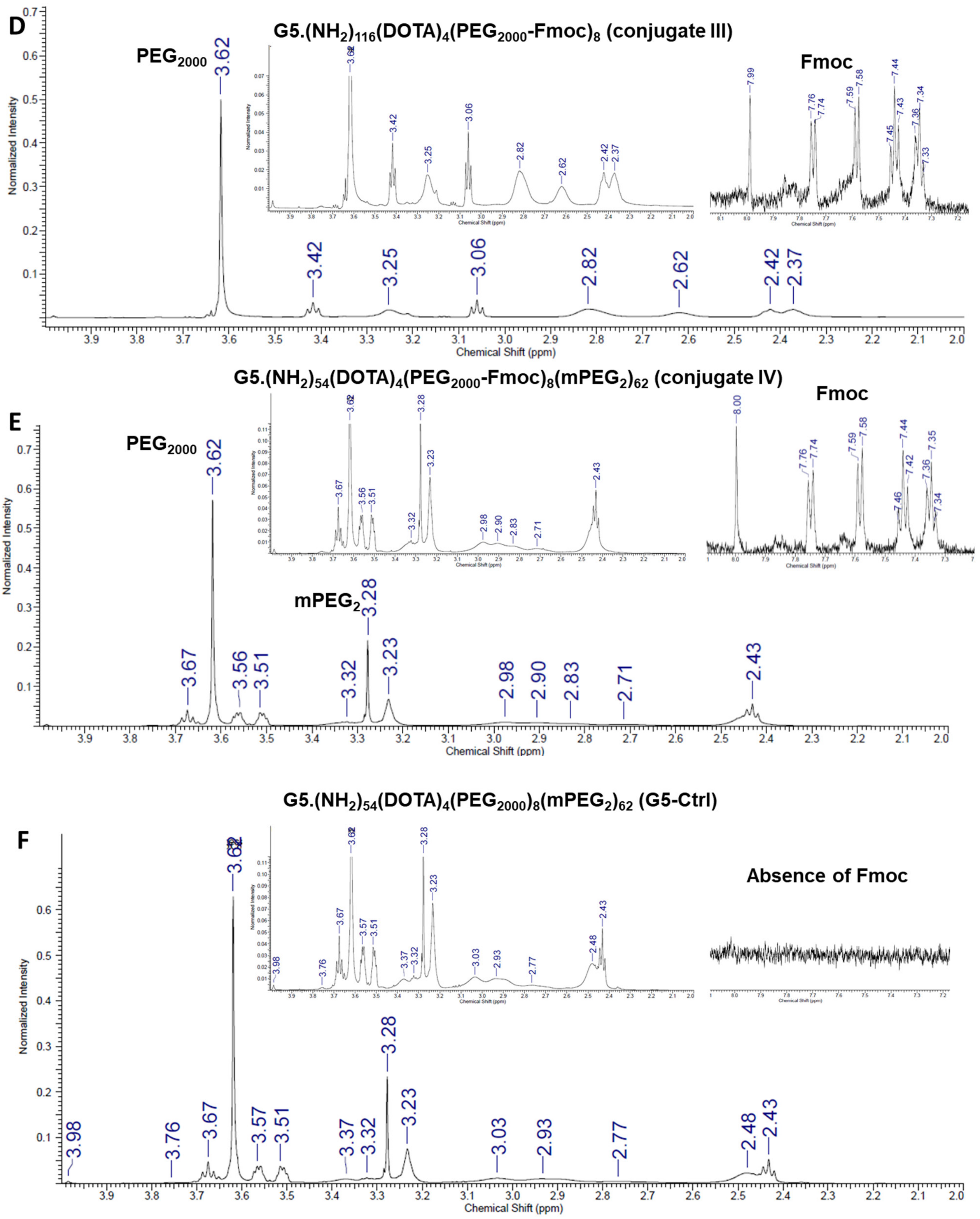

Figure S3. Proton-NMR study. **A** and **B**—^{1}H NMR spectra of the initial $G5(NH_2)_{128}$ dendrimer. Insert - partial structure of PAMAM dendrimer (n = 4) showing theoretical number of CH_2 groups. Assignment of the signals was done based previously reported data [2] and values of integrals are in good agreement with the number of ^{1}H in each group. **C**—^{1}H NMR spectrum of $G5(NH_2)_{124}(DOTA)_4$ (conjugate 2, Figure 1 reaction scheme) showing broadening and shift of the dendrimer signals in particular related to protons V and VI that are close to terminal NH_2 groups and 2 additional peaks at 3.05 and 3.5 ppm upon conjugation of on average 4 molecules of DOTA with $G5(NH_2)_{128}$ dendrimer. **D**—^{1}H NMR spectrum of $G5(NH_2)_{116}(DOTA)_4(PEG_{2000}\text{-}Fmoc)_8$ (conjugate 3, Figure 1 reaction scheme) demonstrating dominant peak related to PEG_{2000}-Fmoc at 3.62 ppm and additional peaks of Fmoc in the aromatic region (insert) and significant broadening of

dendrimer signals. **E**—^{1}H NMR spectrum of G5$(NH_2)_{54}$$(DOTA)_4$$(PEG_{2000})_8$$(mPEG_2)_{62}$ (conjugate 4, Figure 1 reaction scheme) showing additional strong intensity signal at 3.28 ppm related to $mPEG_2$ and further broadening the other peaks. Due to the presence of broad and overlapping signals, their assignment is not feasible. **F**—^{1}H NMR spectrum of G5$(NH_2)_{54}$$(DOTA)_4$$(PEG_{2000})_8$$(mPEG_2)_{62}$ (G5-Ctrl, Figure 1 reaction scheme) indicating deprotection of PEG_{2000} by the absence of peaks in the aromatic region. All spectra were recorded using D_2O as a solvent and a Bruker Avance III 500 MHz NMR spectrometer.

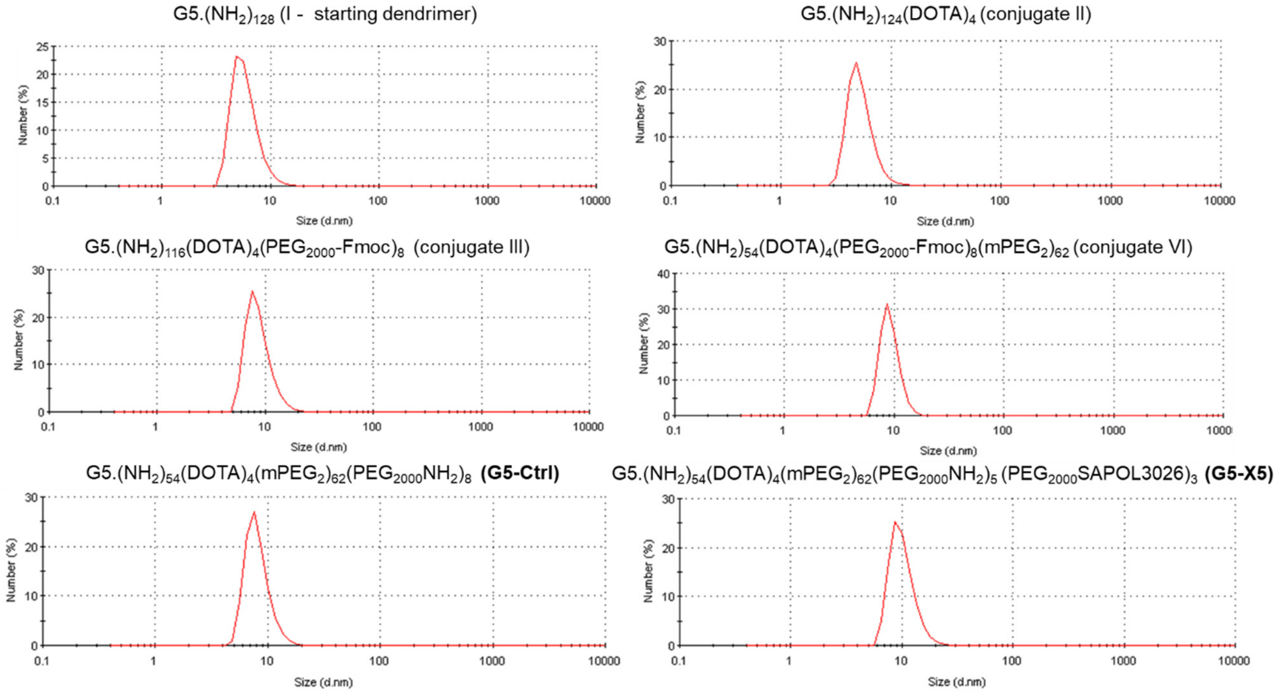

Figure S4. Representative number weighted size distribution acquired for starting dendrimers and all synthetized conjugates, demonstrate an increase in dendrimer size after PEGylation.

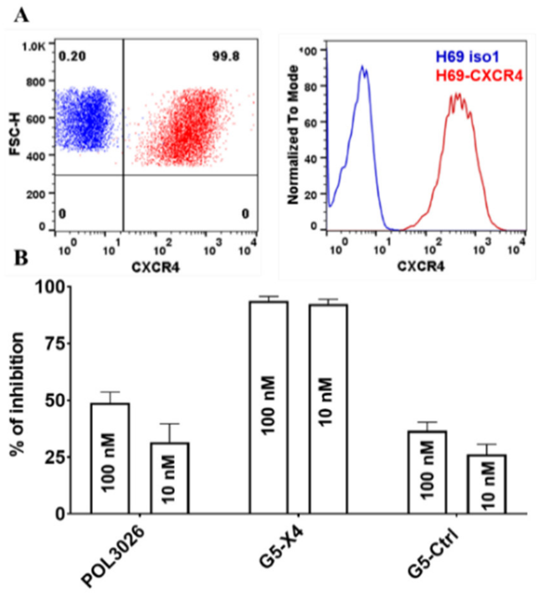

Figure S5. A—Evaluation of CXCR4 expression in H69 small cell lung cancer (SCLC) cell line by flow cytometry. **B**—*In vitro* migration of H69-CXCR4 SCLC cells carried out using a CytoSelect cell migration assay (Cell Biolabs, Inc. San Diego, CA) according to the manufacturer's protocol.Results indicate high CXCR4 expression in H69 cells and superior inhibition of their chemotaxis by G5-X4.

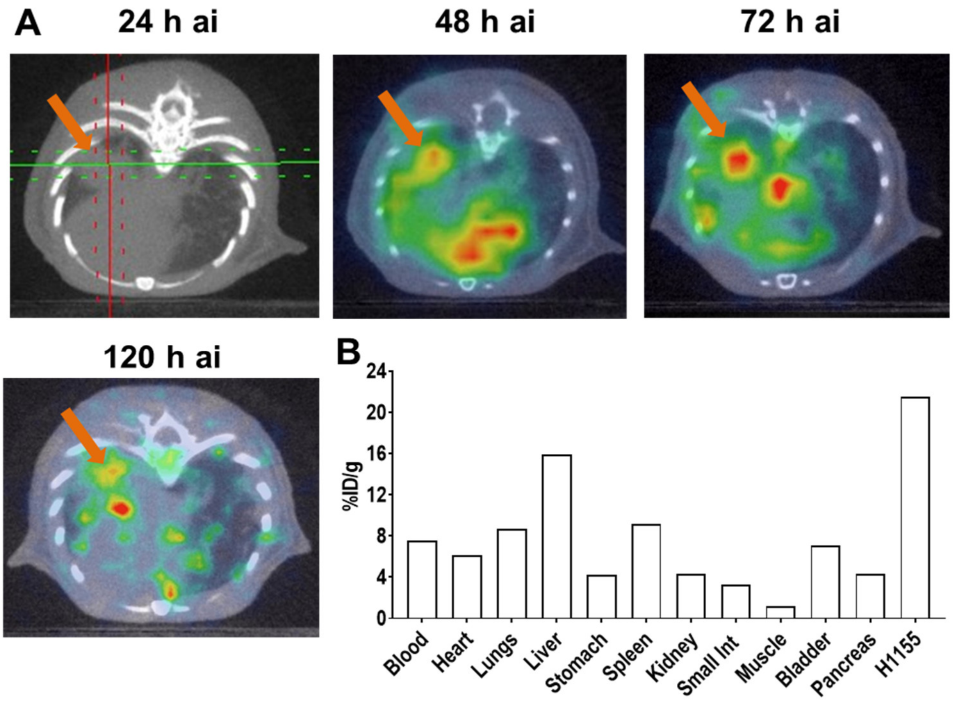

Figure S6. Evaluation of [^{111}In]G5-Ctrl in orthotopic H1155 non-small cell lung cancer (NSCLC) mouse model. **A**—Transaxial *in vivo* SPECT/CT images of a mouse bearing an orthotopic H1155 NSCLC tumor (arrow) that was injected with 350 μCi of [^{111}In]G5-Ctrl. Images were acquired 24, 48, 72 and 120 h after injection (ai). **B**—*Ex vivo* biodistribution of [^{111}In]G5-Ctrl in tissues obtained from the imaged mouse after completion of SPECT/CT imaging. Results indicate relatively high passive accumulation of [^{111}In]G5-Ctrl in NSCLC tumor.

References

1. Lesniak, W.G., et al., Evaluation of PSMA-Targeted PAMAM Dendrimer Nanoparticles in a Murine Model of Prostate Cancer. *Mol. Pharm.*, **2019**, 16(6), 2590-2604.
2. Shi, X.Y., et al., Generational, skeletal and substitutional diversities in generation one poly(amidoamine) dendrimers. *Polymer*, **2005**, 46(9), 3022-3034.
